# Supplementary figures and images for: Warm Blood Meal Increases Digestion Rate and Milk Protein Production to Maximize Reproductive Output for the Tsetse Fly, Glossina morsitans
Source: Insects. 2022 Oct 31;13(11):997. doi: 10.3390/insects13110997 (PMC9695897; doi:10.3390/insects13110997)

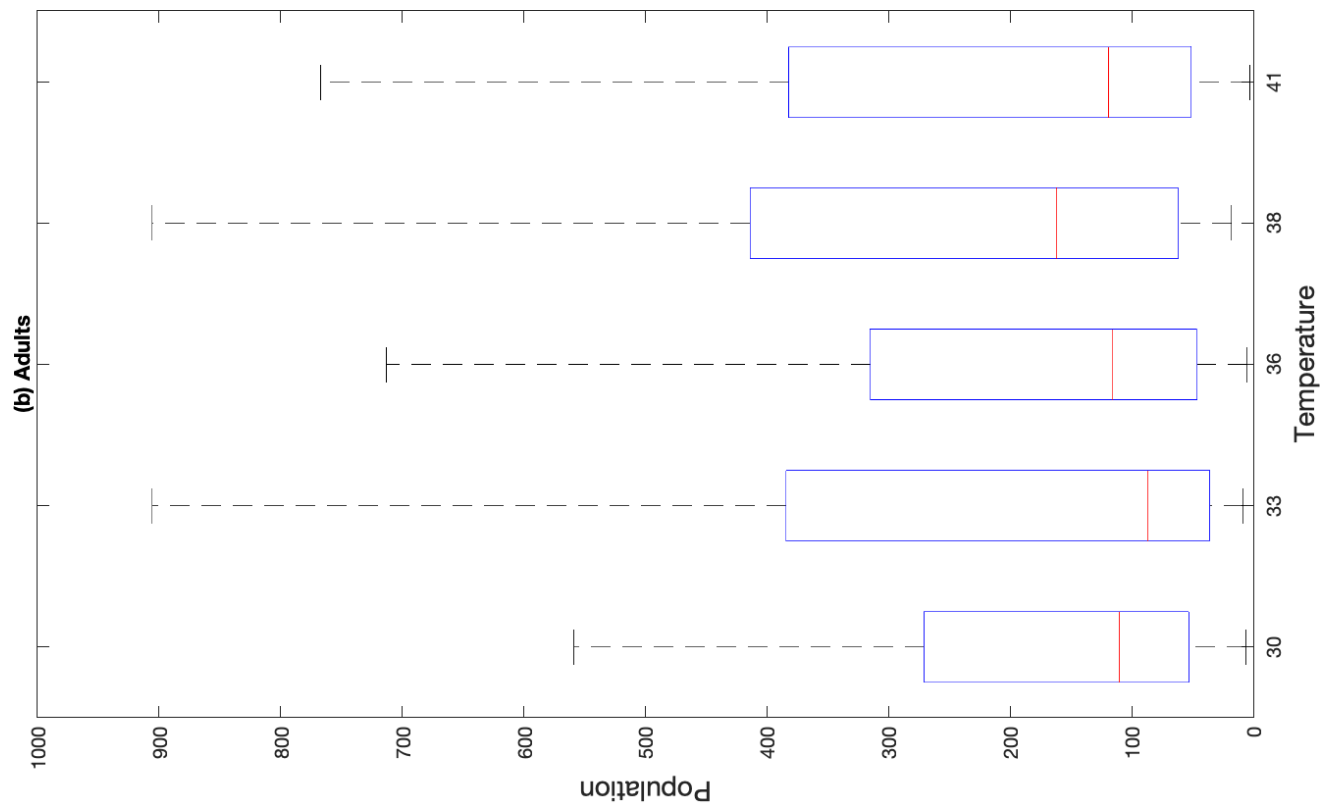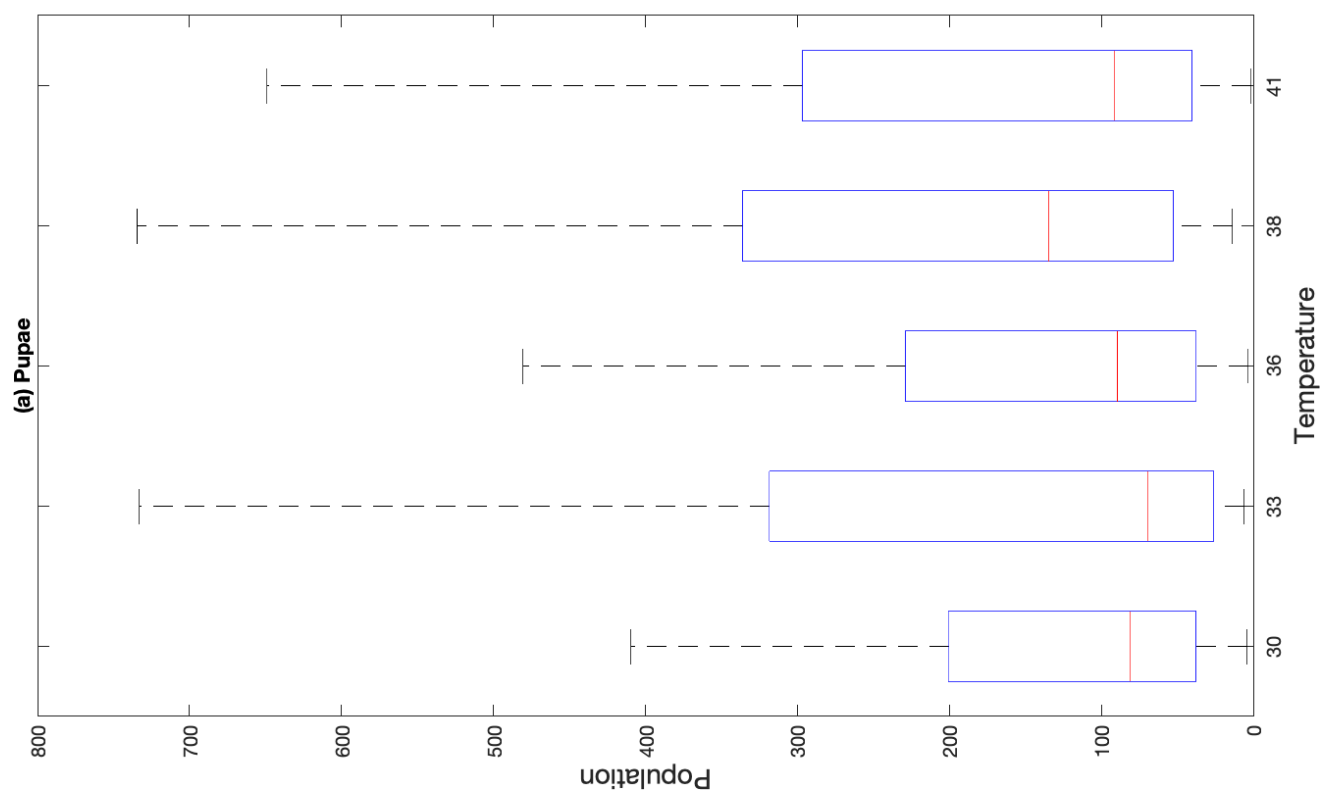

Figure S1. All temperatures values of consuming cool, warm, and hot blood.

Supplement: Supplementary file 1 [file insects-13-00997-s001.zip › insects-1969769-supplementary.pdf]
